# Table Tennis Track Detection Based on Temporal Feature Multiplexing Network

**DOI:** 10.3390/s23031726

**Published:** 2023-02-03

**Authors:** Wenjie Li, Xiangpeng Liu, Kang An, Chengjin Qin, Yuhua Cheng

**Affiliations:** 1 College of Information, Mechanical and Electrical Engineering, Shanghai Normal University, Shanghai 201418, China; 2 School of Mechanical Engineering, Shanghai Jiao Tong University, Shanghai 200240, China; 3 Shanghai Research Institute of Microelectronics, Peking University, Shanghai 201203, China

**Keywords:** motion trajectory, deep learning, object detection, lightweight network, Transformer model, feature reuse

## Abstract

Recording the trajectory of table tennis balls in real-time enables the analysis of the opponent’s attacking characteristics and weaknesses. The current analysis of the ball paths mainly relied on human viewing, which lacked certain theoretical data support. In order to solve the problem of the lack of objective data analysis in the research of table tennis competition, a target detection algorithm-based table tennis trajectory extraction network was proposed to record the trajectory of the table tennis movement in video. The network improved the feature reuse rate in order to achieve a lightweight network and enhance the detection accuracy. The core of the network was the “feature store & return” module, which could store the output of the current network layer and pass the features to the input of the network layer at the next moment to achieve efficient reuse of the features. In this module, the Transformer model was used to secondarily process the features, build the global association information, and enhance the feature richness of the feature map. According to the designed experiments, the detection accuracy of the network was 96.8% for table tennis and 89.1% for target localization. Moreover, the parameter size of the model was only 7.68 MB, and the detection frame rate could reach 634.19 FPS using the hardware for the tests. In summary, the network designed in this paper has the characteristics of both lightweight and high precision in table tennis detection, and the performance of the proposed model significantly outperforms that of the existing models.

## 1. Introduction

Table tennis as an international sport that has a strong base of enthusiasts and is gaining popularity in more and more countries. In table tennis matches, the balls have different trajectories through various strokes, such as chipping, rubbing, snapping, etc. Each player has a personal style of stroke, so it is possible to analyze the trajectory of the ball to determine the attacking and defending style of the opponent in the match [1]. The current method of analyzing the opponent’s trajectory was mainly by watching videos of the opponent’s past matches, which was time-consuming and could not enable obtaining the specific data [2,3]. This paper presents a target detection algorithm for identifying table tennis and determining its position on the table, with the goal of enhancing the ability of players and coaches to analyze the trajectory of the ball.

Recently, significant advancements have been made in computer vision utilizing deep learning techniques, particularly in the area of target detection. Convolutional neural networks are capable of automatically extracting target features, compensating for the disadvantages of traditional detection algorithms that rely on a priori experience to design the feature detectors manually. Compared with the manually designed feature detectors, the features extracted by convolutional neural networks have much better fits to the actual target. Many teams had recently proposed several ball detection solutions that were based on convolutional neural networks. For example, Cao et al. [4] developed a badminton detection scheme based on the Tiny You Only Look Once (YOLO) v2 network in their badminton robotics project. To achieve an efficient detection of targets, they optimized the network structure of Tiny YOLO v2 so that the network could retain semantic information for more small targets during the feature extraction, and the loss function was modified to improve the computational speed. However, the lightweight operation of the network weakened the ability for feature extraction and decreased the detection accuracy compared to that before the optimization. In addition, a camera-based basketball score detection method was presented by Fu et al. [5], the key technology of which was to detect the basketball. A detection algorithm utilizing YOLO was employed to pinpoint the location of the basketball hoop, followed by a motion detection technique based on the frame difference to identify any movement within the hoop’s vicinity, ultimately determining the basketball score. However, the network required the processing of multiple frames of data and needed high performance hardware support to achieve real-time results. In studying the table tennis robot vision system for the detection of table tennis, Zhang et al. [6] presented a quick detection approach that incorporated a multi-color model, which combined the Red-Green-Blue (RGB) color model and the Hue-Saturation-Value (HSV) color model to transform the table tennis region in the image into a binary part, and to locate the center of the ball by calculating the center of the detection frame. On this basis, a Region of Interest (ROI) technique based on the position of the previous frames was proposed for real-time tracking, which used traditional computer vision algorithms in the detection process and a deep learning scheme in the tracking process, so the algorithm’s integration was somewhat lacking. The above cases provided some feasibility arguments for using the target detection algorithm to detect and locate table tennis in this paper. However, there were some disadvantages in using other algorithms for the scenario described in this paper, mainly the following two: firstly, the network was not lightened, and the model was large, which were not suitable for the real-time detection scenarios; secondly, table tennis is smaller in size compared with other sorts of balls, so the percentage of the miss detection is greater. A comparison of various table tennis detection and tracking methods can be found in Table 1, which lists the pros and cons of each network. There was a certain gap between the performance of the previous network and that of the constructed network in this article.

**Figure 1:**
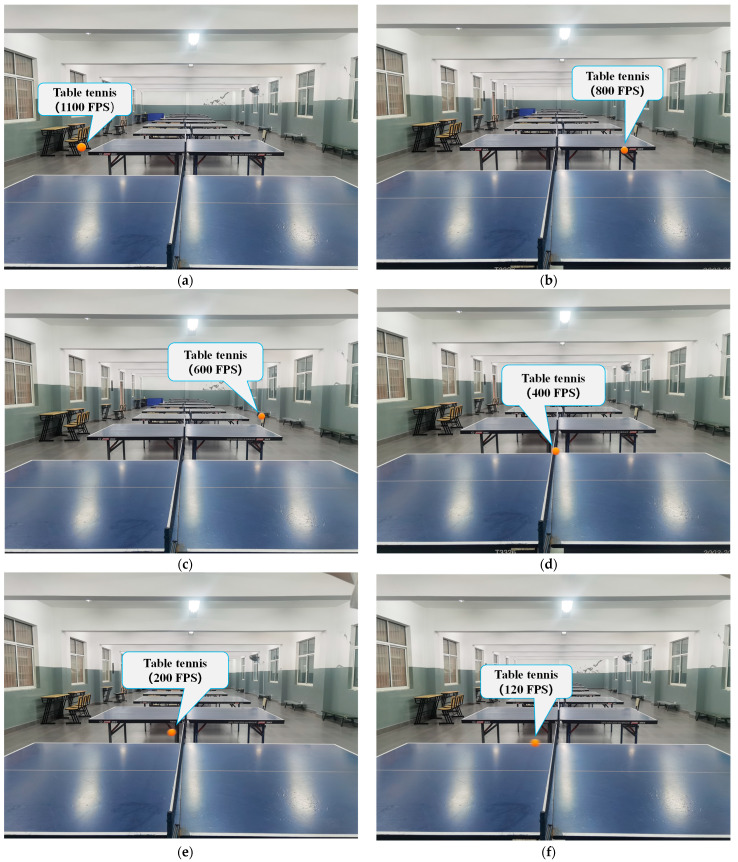
Sample image frames: (**a**) 1100 FPS, (**b**) 800 FPS, (**c**) 600 FPS, (**d**) 400 FPS, (**e**) 200 FPS, (**f**) 120 FPS.

In addition, the core objective of the table tennis trajectory detection task described in this paper is to detect the table tennis and record its position information in consecutive frames. As there was only one table tennis ball on the table during the game, we assign only one target Identity Document (ID), and the detector could be directly applied to the initial image and record the target’s position information without the need to additionally use a re-identification network to discriminate the target’s ID, making the whole network streamlined design. A fixed camera and table setup allowed the detection algorithm to analyze each frame in the video, capturing any changes in the position of the ball. By concatenating the coordinates from the sequence of frames, the movement of the ball could be tracked, and its trajectory could be determined. Regarding the physical feasibility analysis, the average speed of the table tennis during a match was 42 km/h, and the one-way movement time of a table tennis ball was 0.52 s [12]. In this experiment, a super high-speed camera with a maximum frame rate of 1100 FPS was employed to record the actual hitting of the table tennis ball, which was capable of capturing a moving object with a maximum speed of 540 km/h and could meet the requirements for capturing the trajectory of the table tennis ball during a match. In this paper, different frame rates were set to capture the table tennis ball in action, and the resulting frames at different frame rates are shown in Figure 1.

As shown in Figure 1, when the frame rate was higher than 600 FPS, the table tennis had no shadow and was regular. When the frame rate was set to 400 FPS, the ball had tiny shadows and changed slightly from a circle to an oval shape. The position of the sphere could not be accurately located. Therefore, a minimum frame rate of 600 FPS was required to obtain a clear picture of the sphere.

In summary, to overcome the obstacles in table tennis ball detection tasks, this study designed a dedicated detection network, which was able to reduce the use of network layers by efficiently reusing the feature information to achieve the lightweight requirements. The implementation of the network mainly relies on the feature reuse module, which stores the feature information extracted in the previous iteration and passes it to the corresponding network layer in the next iteration, so that the feature information can be fully extracted with only a few network layers [13,14,15]. At the same time, the Transformer module was added to the network to utilize its excellent capability of global feature information extraction, while combining with the local feature extraction capability of the convolutional network, in order to improve the networks’ capacity of precepting small targets [16,17,18,19]. Furthermore, the positional information exported by the detection module is likewise multiplexed. Because of the high similarity of the adjacent frames and the small moving position of the table tennis ball, the position coordinates of the table tennis ball in the current frame can be obtained by fine-tuning the coordinates based on the position prediction results of the previous snapshot and combined with the feature information of the current frame.

## 2. Related Work

Computer vision technology is broadly used in sports. The related algorithms have gradually changed from the traditional target detection algorithms to deep learning-based target detection solutions. In the sports events field, there are many excellent cases for early solutions using image processing for the detection of specific targets. For example, Fang and Sun [20] had researched human motion recognition techniques in the images of a dancing video. They proposed a new cfrdF method based on example templates, which used Cascading Style Sheet (CSS)-based human feature similarity as a feature, then devised an adaptive angle and continuous-scale spatial matching algorithm, and finally calculated the similarity between the horizontal plate and the detected image value; after setting the threshold value, the region of the human body could be matched. Moreover, they designed the adaptive pattern function to extract human motions from the videos and photographs of dance sport using the self-similar structure of the human and the object color as the fundamental characteristics. However, the primary drawback of this way was that the extraction of human movements mainly relied on the human body and objects, which could cause false recognition when there were blocks of pixels with similar color gamut around the human body, resulting in non-standard extracted movements. Pan and Li [21] proposed a novel recognition pipeline to recognize the human motion in basketball scenes. In this detection process, an affinity graph was first constructed to obtain the motion regions, and entropy within the motion regions was calculated to extract the motion blocks. After that, the features were calculated from the motion block using a Gaussian Mixture Model (GMM) and the key frames were selected by modeling the variation between the adjacent frames, then the gradient histogram was employed to indicate the pose description operator of the basketball action. Finally, they used the linear combination of motion block features, pose description operators, and the K-Nearest Neighbor (KNN) algorithm to achieve the basketball action recognition. Nevertheless, for the extraction of the video motion region, the extracted motion region was missed, due to multi-target occlusion or background environment interference, which affected the subsequent detection accuracy. Sun et al. [22] used an unsupervised clustering method to design a classification strategy to achieve fast target detection, being able to cope with environmental illumination changes, and improve the recognition accuracy of athletes in the sports fields. In this scheme, the AdaBoost algorithm was optimized in terms of both the fast feature selection and dual-threshold judgment. Furthermore, for the human target with complex human pose and partial occlusion, they proposed to represent the physical body by many parts to solve the human recognition problem under complex background and occlusion conditions. The advantage was that the whole individual is represented by a part of the human body. However, when the capturing distance was long and the target was small, its local features were not obvious enough, so there were serious cases of missing detection. Mei and Wang [23] detected and tracked the moving objects in the sports videos based on the Scale Invariant Feature Transform (SIFT) algorithm. The plan split the point cloud data into numerous cubic grids using its coordinate system, found each grid’s center of gravity, and then replaced all of the grid points’ coordinates with that of the center of gravity. The coordinates were used to mark and record the athlete’s motion trajectories. The prerequisite of this solution lied in the acquisition of the point cloud data, for which the data acquisition had some limitations in use, due to the harsh conditions. For athlete target tracking, Ong’s team [24] used the Flower Pollination Algorithm (FPA) to track athletes in the video, which initially used a search interface with the athletes’ center-of-mass coordinates, search window width, and length properties to indicate the athletes’ current positions. Afterwards, in order to identify the athletes’ many promising positions in one frame, the histograms of the region within the search window were evaluated using the HSV. The barotropic distance between the HSV histogram of the athlete’s initial position and their possible position in the current frame was then calculated, which in turn enabled the tracking of the target. This method mainly relied on the color property of the search window, which functioned as the basis for determining the target tracking. When the color was too complex or monotonous, it would cause the frame screen to be missed and mis-checked, which resulted in frequent changes of the target ID during the tracking process and a poor tracking effect. In summary, for the traditional algorithms, they relied more on the pixel-to-pixel difference of the image to achieve the determination of the target, which also made the algorithm poor in robustness and easily disturbed by the external environment.

Recent years have seen the explosive growth of target identification systems based on the neural networks of deep learning, which relied on efficient network learning capabilities, and had gradually replaced the traditional approaches and enabled the wider application of target detection algorithms in sports [25]. For instance, in the task of basketball motion detection, Liu et al. [26] used the high-resolution capability of the convolutional neural networks to extract images, and then computed pre-processing tasks for the identification of each image of human motion in the video stream. Afterwards, the Long Short-Term Memory (LSTM)-based skeleton recognition algorithm was employed to test the critical points of the human body. The method utilized the convolutional network to extract the target in the image, which had significantly improved the accuracy, compared with the traditional models using the color field extraction, but it was only able to judge the action of a single target. To improve this model, in the task of motion pose analysis, Sun and Li [27] presented a motion training-based pose analysis approach on the ground of a multi-scale spatiotemporal map convolutional network. The first step in the method is to create a spatiotemporal representation of the skeleton, followed by applying a convolutional operation on the resulting image. Lastly, the convolution results were fused with linear weights to capture the features of the different time length action types. Through the analysis of the experimental data, the main drawback was the imperfect feature processing for the long time sequences. When the video was very long, the front-end features could not build sufficient correlation, which made the accuracy of judging for the action decrease. For motion target tracking, Duan [28] applied a deep neural network to multi-target the motion tracking in the sports video. After the target frame selection was determined, the detector scanned each motion video frame one after another, and then observed the previously discovered subregions and learned image frames until the current moment could highly resemble the target to be tracked. In addition, the remote sensing images that were pre-processed were transformed into grayscale images, and their histograms were adjusted to a standard scale. In addition, a combination of the region growth function was applied to choose the appropriate height threshold, which was conducted with the goal of eliminating the motion shadows from multiple targets in the sports videos and creating a multi-target network model. The method had great improvements in target detection, but it still adopted the traditional architecture in tracking, resulting in the poor overall performance. Huang [29] proposed a method for accurately recognizing continuous motion action using deep learning to enhance the recognition performance. The creation of the map of the motion pose sequence was achieved by utilizing a neural network to predict the primary joints and the central vectors of a person in a grid layout. The human main points were then grouped based on how far they were from the human center. After that, the motion pose sequence diagram was inputted into the deep learning network, and the optimal strategy for continuous action recognition was applied to obtain the accurate recognition of continuous motion poses. However, this method required more computational resources for inference computation due to the complexity of the convolutional approach, which was not ideal in terms of the real-time computation. Baclig et al. [30] used an autonomous learning-based convolutional neural network for the human pose estimation, detecting and identifying squash players’ position data in the court; this model used inverse perspective mapping to convert the pixel coordinates into the court coordinates and to locate the players’ coordinate information in the court. The method had a high accuracy for the athlete target detection, but the acquisition of the position information was based on the coordinate information obtained from the detection. The detection had some errors, which would be amplified in the subsequent calculation and influence the positioning accuracy. In conclusion, the deep learning-based detection algorithm had a large improvement in the detection accuracy of the target compared with the traditional algorithms, and the automatic parameter optimization of the neural network enabled stronger robustness.

In addition, many schemes were proposed for the optimal design of network structures in the convolutional neural networks. For example, Shah et al. [31] proposed to use TabNet with spatial attention to extract the spatial information for hyperspectral image classification, in which 2D Convolutional Neural Networks (CNN) were incorporated into an attention converter for the spatial soft feature selection. Additionally, the dynamic texture smoothing approach was used to build the structural contour during the pre-processing step, where the matrix was utilized via extracting features. The retrieved SP was then fed into the TabNet to further improve the performance. Qin et al. [32] proposed a new multiscale CNN-LSTM neural network named MSCNN LSTM Net, which had a residual CNN denoising module. To begin with, a specific residual CNN structure was created for the purpose of eliminating noise from the original signal. Additionally, a novel loss function was developed by incorporating a residual loss component, and then a multiscale convolutional neural network block was created to execute the multiscale feature extraction, while taking the key characteristics of the original signals at various scales into consideration. In addition, a variety of branches featuring different convolutional kernel sizes were employed in multiple convolutional layers to extract the features of various time-scales, thereby increasing the robustness of the model. Hou et al. [33] proposed a lightweight network architecture algorithm which is derived from MobileNetv3-YOLOv5s (MR-YOLO). As the starting procedure, the MobileNetv3 structure was employed to substitute a portion of the backbone network in YOLOv5s for feature extraction, with the goal of reducing the size and computation time of the network. Additionally, a CSPNet cross-level local network was incorporated to ensure high accuracy, while minimizing the computational effort. In order to increase the positioning accuracy, the focus loss function was reformed, and boundary box regression rate was increased. Finally, the rotational angle method was added by improving the initial framework design and the bounding box regression method of the YOLOv5 target detection network. Munteanu et al. [34] trained and compared three deep learning models, namely YOLOv5, Single Shot MultiBox Detector (SSD), and EfficientDet. The newly generated system showed high accuracy in the object recognition tasks. Liu’s team [35] proposed an enhanced multi-headed self-focused convolutional neural network (EMSACNN) with a two-stage feature extraction model. The architecture employed the utilization of multi-headed self-attentive modules as the primary feature extractor to completely extract the correlation of the input, followed by the utilization of a two-dimensional CNN (2dCNN) as the secondary feature extractor. The actual data were used to validate the proposed EMSACNN’s accuracy and efficiency.

Meanwhile, the research on convolutional neural networks had also taken a new direction in recent years, in which the Transformer model in the field of Neuro-Linguistic Programming (NLP) was modified and transplanted to convolutional neural networks. The Transformer model was also added to the network, in comparison to the normal convolutional neural networks, to make up for the convolutional model’s failure to build relationships between features, which led to the incapability of fully utilizing the contextual feature information and low sensitivity to the global information [36,37,38]. The Transformer model was originally proposed by Google [39] and applied to the field of natural language processing to solve the problem of establishing the long contextual dependencies using the recurrent neural networks. The Transformer model in natural language processing used an attention mechanism to establish the connection between data from two arbitrary locations to obtain the long-range information. Therefore, in subsequent studies, scholars had continuously migrated the Transformer model to the computer vision domain to further improve the network performance [40,41,42,43]. In addition, there were many practical applications in the field of target detection. Dai’s et al. [44] proposed a two-stage Transformer network for the task of pancreatic segmentation in Computed Tomography (CT) images. In this network, the coarse segmentation was first performed using 2D Unet to create potential regions of the pancreas. Furthermore, in the following stage, the deformable convolution was integrated into the Vision Transformer for the fine segmentation of the pancreatic region. As for other medical segmentation tasks, Yang [45] presented the TSE DeepLab network structure. Based on the DeepLabv3 framework, the network retained the original null convolution for extracting local features and converted the post-backbone feature maps into the vision tokens, which were further fed into the Transformer module to enhance the global feature extraction capability. At the same time, squeezing and excitation components were added after the Transformer module to rank the channel importance, so that the model focused on the important pixel features of each channel to improve the segmentation accuracy. Zhang [46] proposed a new CNN-Transformer hybrid structure based on APT-Net, a medical image segmentation network. In order to deliver more accurate position information for the token sequences in the Transformer, the network incorporated an adaptive position encoding module for combining the position information from several sensory fields. Moreover, the dual-path was parallelly decoded with basic and guided information paths, and it simultaneously processed the multi-scale feature maps to effectively utilize the contextual information for segmentation. The above three networks [44,45,46] applied the Transformer model to the segmentation task, which mainly obtained the global information of the image through the Transformer model. However, the processing was performed by the tokens in the Transformer module, making the correlation of the global feature map not strong, and there were some problems in the feature relationship loss. As for the enhancement of the tokens’ connection, there were typical Swin Transformer models. For instance, Wang [47] investigated a framework based on the feature localization approach of the deep point cloud series; it employed the classical encoder-decoder structured Swin-T-NFC CRFs for both the point cloud super-resolution images and image semantic segmentation. The architecture consisted of a Swin Transformer-based encoder, as well as a decoder grounded on fully connected conditional random fields (FC-CRFs) cascaded by a pyramidal pooling module and numerous jump connections. In addition, the shift window policy was connected with the encoder and decoder, allowing for cross-window interactions. As a consequence, patches from various feature map windows might take part in the self-attentive computation, enhancing the modeling capabilities.

**Figure 2:**
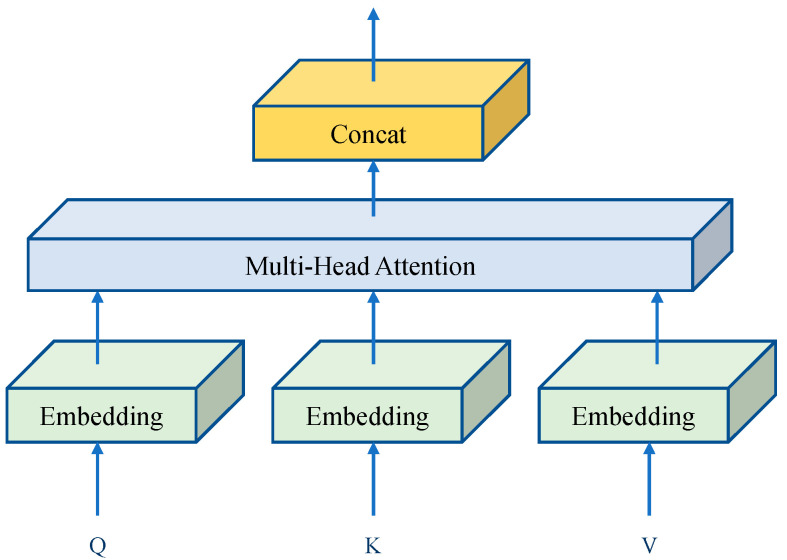
The structure of Transformer model.

To summarize, the Transformer model had been adopted in the domain of computer vision. The detailed computational process for the Transformer module applied in computer vision is shown below. The calculation process of the Transformer model differed from the structure of the model used in natural language processing, mainly in that the data accepted by the Transformer model was one-dimensional vector, so the use of Transformer in the field of computational vision needed to perform preprocessing operations [48,49,50,51]. Firstly, the feature map with parameter size $x\in R^{H\times W\times C}$ was equally divided into a sequence of feature blocks $x_{p}\in R^{N\times (p^{2}\times C)}$, where *p* was the length and width of the feature blocks and *N* was the number of feature blocks partitioned from the feature map. Then, each feature block was mapped $p^{2}\times C$ to *D* dimensions by a linear transformation $E\in R^{(p^{2}\times C)\times D}$ to obtain $x_{p}E\in R^{N\times D}$, with each one-dimensional feature block represented as a token [52,53,54]. Afterwards, the tokens were fed into the Transformer model; the internal computational structure of the Transformer was a multi-layered, parallel computation, primarily comprising of two sections, the first being a multi-headed self-attention layer and the second being a multi-layer perceptron [55,56], as shown in Figure 2.

In contrast, the extraction of the long distance-relationships for the feature information was obtained by multiple self-attentive calculations, which were essentially multiple parallel self-attentive calculations [57,58,59], each expressed as:

(1) $$SA=Softmax(\frac{QK^{T}}{\sqrt{d_{k}}})V$$

where *Q*, *K*, and *V* were feature vectors obtained from the input one-dimensional sequence token after the mapping function, *Softmax* was the activation function, and $d_{k}$ was the number of one-dimensional vectors obtained from the transformation of the feature blocks divided into the sub-feature maps. The multi-headed self-attentive computation was acquired by fusing the results of multiple self-attentive computations obtained in parallel:

(2) $$MSA=\left[SA_{1},SA_{2},\ldots ,SA_{n}\right]$$

where $SA_{i}$ (*i* =*1*, *2*, ..., *n*) were the self-attentive weights of the *i*th feature block calculated by Equation (1).

In conclusion, the Transformer model had the following advantages over the convolutional models: firstly, it constructed long-distance feature relationships, and the Transformer used the attention mechanism to obtain the contextual information of the feature map, which made up for the slow process of expanding from the local features to the global features through layer-by-layer down sampling operations [60,61,62]. Secondly, it had the capability of multimodal fusion. The input parameters of the Transformer model were one-dimensional features, which made it possible to input other one-dimensional features such as time and text into the Transformer model at the same time and fused them with the token after the feature map transformation [63,64,65]. Thirdly, it had a stronger learning ability, and the Transformer model used multiple self-attentive mechanisms for the whole feature map to learn, with each self-attention mechanism independently computing the subspace features before merging [66,67].

## 3. Materials and Methods

The difficulty in this detection task was that the detection frame rate of the model needed to be higher than the video frame rate in order to achieve the effect of the real-time detection. Current designs for lightweight networks were mainly applied in the following areas: the first was the lightweight design of convolutional layers, such as deep separable convolution [68,69,70]. The second was the design of convolutional modules, e.g., the annealing module used in Squeeze Net to achieve light-weighting by reducing the network parameters [71,72]. The third was to use searching mechanisms in the network architecture to disconnect invalid neurons and retain the valid connections alone [73,74,75]. All of the above solutions had some drawbacks in this study. The light-weighting of the convolutional layers and convolutional modules was mainly used to reduce the network parameters, but it was still necessary to build the deep convolutional network to extract sufficient feature information [76]. In summary, the design of the lightweight networks was based on improving the feature reuse rate and reducing the number of network layers through efficient feature reuse.

### 3.1. Design of the Main Structure of the Network

In the main structure of the network in this paper, the use of network layers was reduced by increasing the utilization of the features. Therefore, the features of the previous moment were reused in the current moment in the network design of this work, and the network was named as the temporal feature multiplexing network (TFMN) according to this characteristic.

**Figure 3:**
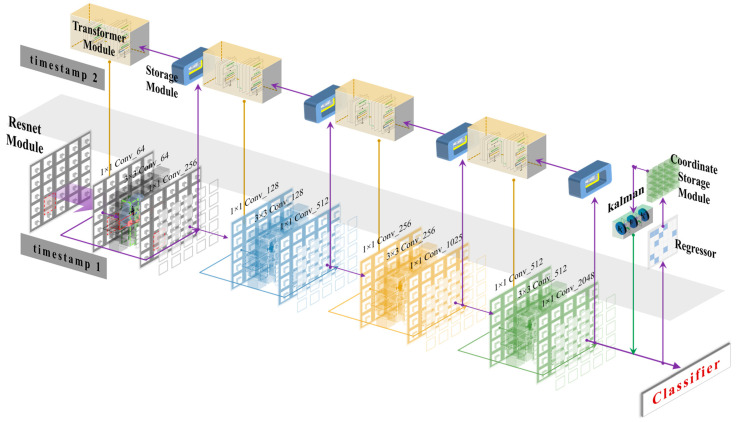
Temporal feature multiplexing network.

As shown in Figure 3, the overall network used six network layers. For the output of each network layer, when it had finished extracting the features, the extracted features were passed to the next network layer and passed to the feature storage module to save the current moment’s extracted feature information at the same time. Regarding the input to the network layer, in addition to the usual output from the upper network layer, the previous moment’s feature information was saved in the storage module simultaneously. The addition of the storage module allowed the feature extraction network to make full use of the feature information, and, as the number of detections increases, more feature information was stored and reused, i.e., the fusion of features in the time dimension achieved the effect of deep network extraction.

### 3.2. Design of Feature Information Return Module

**Figure 4:**
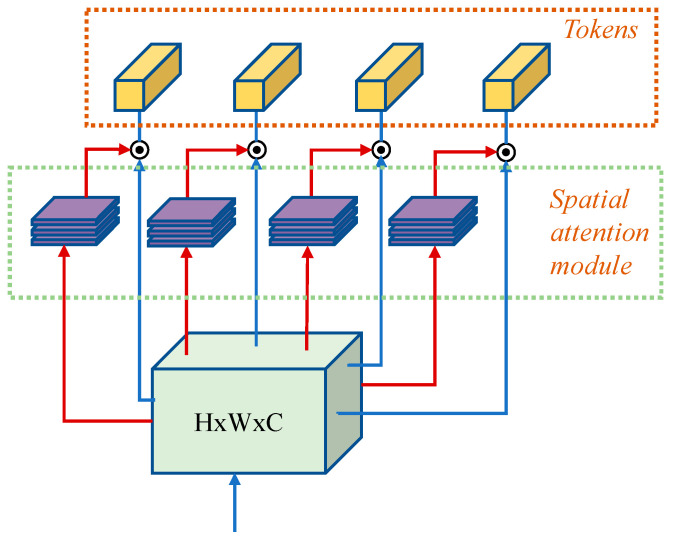
TokenLearner calculation flow.

Due to the small number of layers in the backbone network, only three down sampling operations were performed, so that the feature information extracted from the convolutional network was mainly local feature information. In order to achieve effective feature reuse, this paper added the Transformer model to the feature storage and returned the module, with the aim of computing global attention to the returned features and constructing long-range feature relationships in the feature map to functionally complement the convolutional network. However, due to the large computational size of the Transformer model, adding the Transformer model within each module would lead to a proliferation of the number of parameters in the network and a loss of the advantage of light-weighting. Therefore, this paper incorporated the TokenLearner mechanism within the Transformer model to reduce the computational effort of the Transformer by generating only a small number of tokens from the transformation of the feature map [77]. The computational process of the adopted TokenLearner mechanism is shown in Figure 4.

The input to the Transformer module had a feature map size of $x\in R^{H\times W\times C}$. Unlike the operation of dividing the feature blocks into one-dimensional tokens for the feature map, the TokenLearner mechanism was able to calculate the weight heat map of the corresponding spatial attention using the N group spatial attention mechanism with the input feature map, respectively, and then, the weight heat map was multiplied with the input to complete the assignment operation to obtain the N weighted feature vectors, $z\in R^{H\times W\times C}$. The calculation process could be expressed as follows:

(3) $$z_{i}=A_{i}\left(x\right)=W_{i}⊗x=\alpha_{i}\left(x\right)⊗x$$

**Figure 5:**
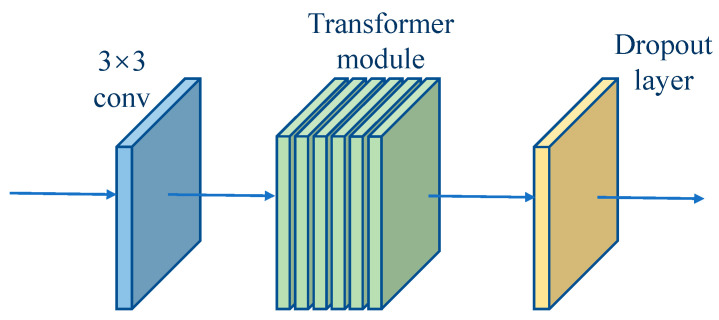
Feature storage and return module.

Afterwards, spatial pooling was used to transform the weighted $z\in R^{H\times W\times C}$ into $z^{\prime }\in R^{1\times 1\times C}$, which stood for tokens. Compared with the standard token acquisition method, TokenLearner was able to determine the number of tokens generated by setting the number of spatial attention computation groups and avoiding the large number of tokens generated by dividing the feature map and long computation sequence [78,79]. In addition, the spatial attention mechanism allowed the model to extract the important regions of the image adaptively. Different from the disadvantage of the poor data correlation caused by dividing the feature map, the feature map could be directly converted into tokens by spatial pooling without segmentation, which effectively ensured the consistency of feature information [80,81,82]. In this paper, six groups of spatial attention mechanisms were set for each Transformer module to extract the network, i.e., the number of tokens input to each Transformer module was four. The detailed structure of the feature storage and return module was shown in Figure 5.

When the features from the previous moment were passed back to the current moment, the 3 × 3 convolutional layer was first used to further extract the features, and then the Transformer module was applied to obtain the global feature information. Finally, the dropout layer was used to prevent overfitting caused by the high similarity of features in the frame sequence when the features were passed back to the backbone network [83,84,85,86].

### 3.3. Design of the Location Information Return Module

**Figure 6:**
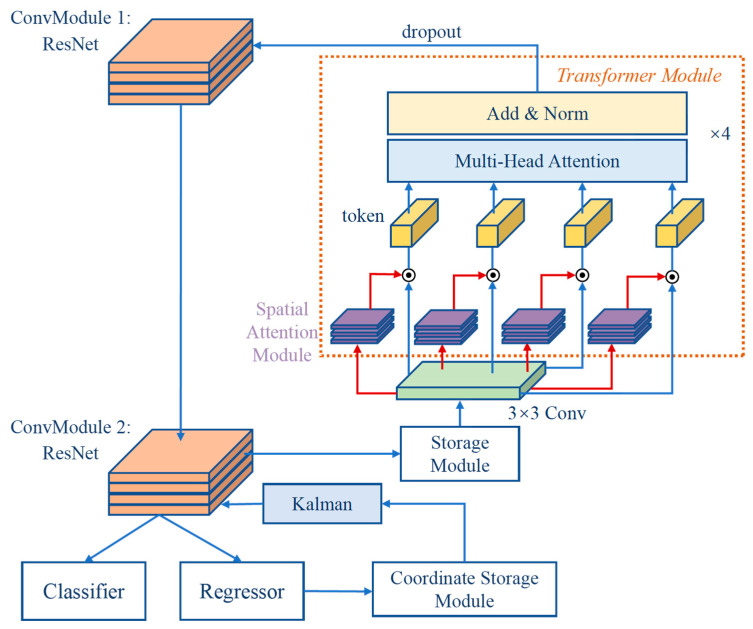
Detailed network structure (partial).

The detector of the proposed model consisted of two parts: the classifier and the position regressor. In the video frames, the table tennis trajectory was continuous and regular, i.e., the table tennis in the current frame was only slightly offset from the table tennis in the previous frame after the video had been drawn (the camera was stationary). Therefore, the target position information detected in the current frame was stored in the network. When the next frame was detected, the stored position information was corrected by the Kalman filter for prediction, where the resulting position was mapped in the feature map and passed to the regressor for the prediction of the target in the current frame. The returned location information parameters were represented using the vector $\left(u,v,\gamma ,h,\dot{u},\dot{v},\dot{\gamma },\dot{h}\right)$, where (u,v) represented the centroid of the target at the previous moment, h denoted the height of the position prediction frame at the previous moment, γ stood for the aspect ratio of the position prediction frame at the previous moment, $\left(\dot{u},\dot{v},\dot{\gamma },\dot{h}\right)$ meant the velocity component of the above four elements, and (u,v,γ,h) was predicted for the current moment using the Kalman filter of the homogeneous and linear observation models [87,88,89,90]. The structure of the cell network after the combination of the optimization modules is shown in Figure 6.

Taking the last dimensional module in Figure 6 as an example, after the convolution module 2 received the feature map output by the convolution module 1 and completed the feature extraction operation, the features were passed to the storage unit to retain the extracted feature information. In the detection of the next moment of the frame, the Transformer module performed the construction of global feature relations to enhance the dominant degree of the target in the frame. In this operation, the stored feature maps of the previous moment were first subjected to feature transition operation by a 3 × 3 convolution, after which four spatial attention sub-modules were set to calculate the associated heat maps. Then, the computed spatial heat weight maps were assigned to the feature maps separately to obtain the four sub-feature maps. In addition, they were transformed into tokens for multi-headed self-attention calculation, and finally the obtained feature maps were fused with the input of convolution module 1 to enhance the richness of the input information. For example, when the convolution module 2 was the last feature extraction module, the output features were utilized by the classifier and regression models to identify the category and location of the target as positive or negative. Furthermore, for the position information output by the regressor, the convolution module 2 was also equipped with a coordinate sub-module. Based on this theory, the stored position coordinates were pre-determined by the Kalman filter for the next position determination. Since the pre-determined position was mapped into the feature map, the regressor could make more accurate position judgments based on the information of position.

## 4. Results Analysis and Discussion

### 4.1. Introduction to Dataset Production and Experimental Environment

**Figure 7:**
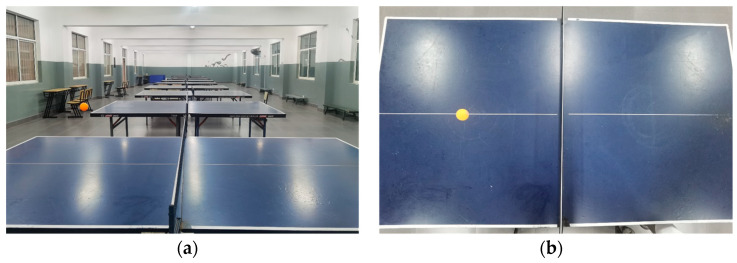
Example of a dataset: (**a**) sample data taken horizontally to the desktop and (**b**) sample data taken perpendicular to the desktop.

As the location information obtained by the detection was relative to the one on the screen, it was not the actual location information. Therefore, in this study, the position information of the ball relative to the table was obtained by capturing horizontally and vertically the overhead relative to the table, so the real position information can be acquired by post-conversion. Based on this principle, 50 video clips of table tennis, with sparring of 5 min each, were taken from two angles and produced as a dataset by frame extraction, as shown in Figure 7.

The experimental environment and network hyperparameter settings used in this experiment are as follows. For the hardware environment, the central processor was Intel i7 11700 and the graphics processor was Nvidia RTX 3070 with 5888 CUDA cores and 184 Tensor cores. Regarding the software, PyTorch 1.11.0 was used as the deep learning framework API, the CUDA computing platform version was 11.3, and the cuDNN computational acceleration library version was 8.2.1. For the main hyperparameters in the experimental environment, the following hyperparameter settings were obtained after several training sessions. The number of epoch iterations was 200, and the initial learning rate was set to 0.001. The original learning rate was 0.001 and gradually converged to 0.00001 after training; the momentum parameter was 0.954; and the weight decay rate was 0.0013.

In this paper, the performance of the network model was evaluated in terms of recognition accuracy, localization accuracy, and model complexity, and the model was analyzed using *Precision*, *Recall*, *AP*, *IoU*, and *Parameter*, as shown in Formulas (4)–(8):

(4) $$Precision=\frac{TP}{TP+FP}$$

(5) $$Recall=\frac{TP}{TP+FN}$$

(6) $$AP=\int_{0}^{1}P_{smooth}\left(r\right)dr$$

(7) $$IoU=\frac{GT\cap PR}{GT\cup PR}$$

(8) $$P=\left(C_{in}\times K^{2}+1\right)C_{out}$$

The performance of the model was measured by calculating various metrics, such as the number of correctly classified positive instances, TP; the number of negatively classified instances predicted as positive, FP; the number of positively classified instances predicted as negative, FN; the position of the marker, *GT* (GroundTruthBox); the position of the model’s output prediction, *PR* (PredictionBox); the number of channels in the input feature map, $C_{in}$; the number of channels in the output feature map, $C_{out}$; and the size of the convolution kernel, K. In addition, this article uses the FPS indicator to assist the model complexity *P* (*Parameter*) indicator. In a more intuitive way, the detection speed of each model using the same hardware environment is expressed.

### 4.2. Ablation Experiments

In the experimental session, an ablation experiment was first designed to disassemble the network model into six networks. The data were compared to determine the effectiveness of each network module in the overall network. Then, five different sets of hyperparameters were set to train the six models in the ablation experiment several times, and the average value was taken as the optimal parameter performance index value for each model. The control groups of the set hyperparameters are shown in Table 2.

**Figure 8:**
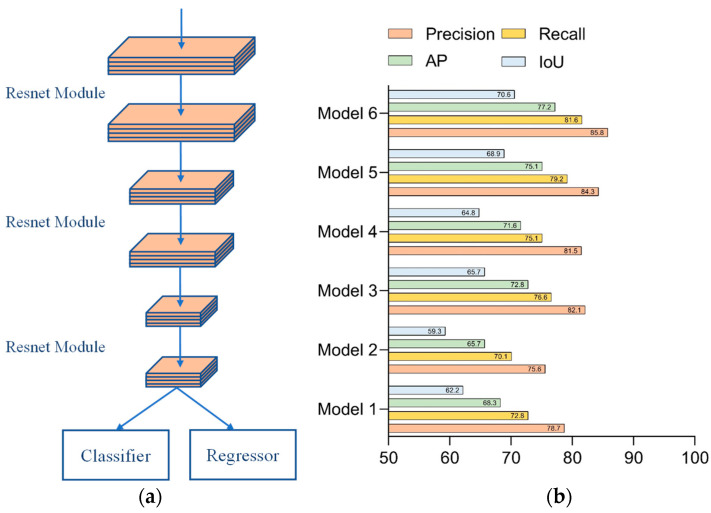
Network 1: (**a**) configuration of Network 1 and (**b**) test results for each parameter of Network 1.

**Figure 9:**
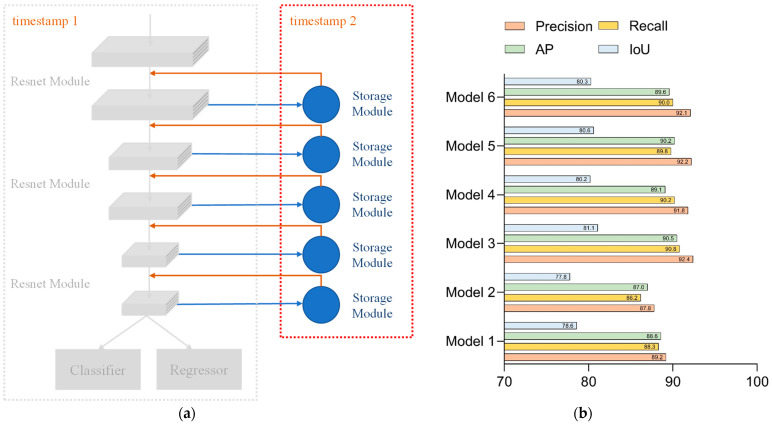
Network 2: (**a**) configuration of Network 2 and (**b**) test results for each parameter of Network 2.

**Figure 10:**
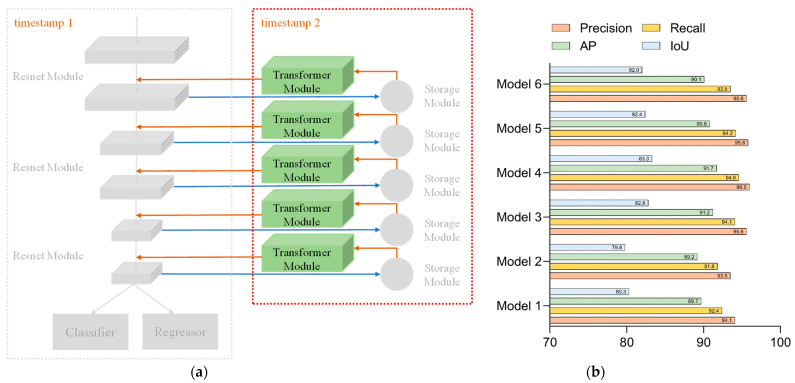
Network 3: (**a**) configuration of Network 3 and (**b**) test results for each parameter of Network 3.

**Figure 11:**
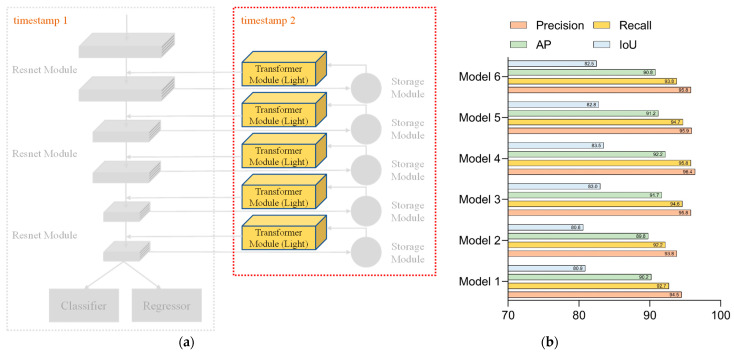
Network 4: (**a**) configuration of Network 4 and (**b**) test results for each parameter of Network 4.

**Figure 12:**
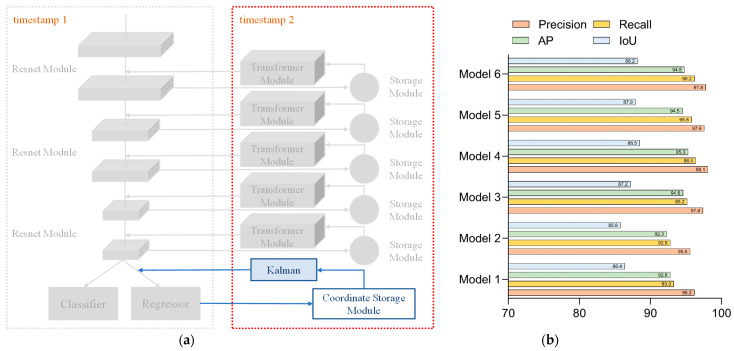
Network 5: (**a**) configuration of Network 5 and (**b**) test results for each parameter of Network 5.

**Figure 13:**
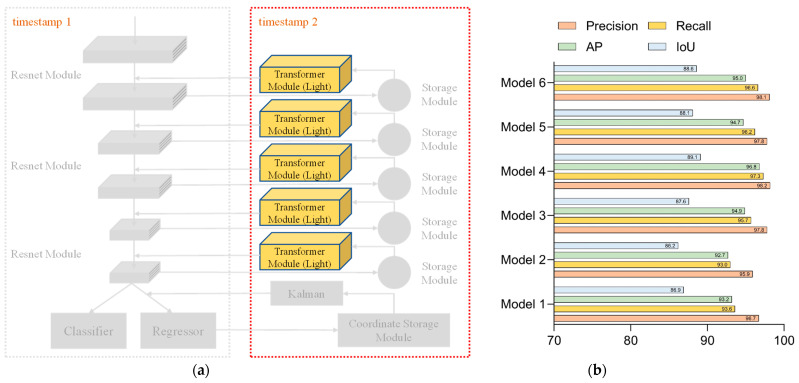
Network 6: (**a**) configuration of Network 6 and (**b**) test results for each parameter of Network 6.

Based on the above set of hyperparameters, six ablation networks were trained and tested, and the network configuration and test results are shown in Figure 8, Figure 9, Figure 10, Figure 11, Figure 12 and Figure 13.

Network 1 consisted of stacks of residual modules alone to form the backbone network. The configuration of Network 1 could be seen in Figure 8a. The test results of each parameter of Network 1 are shown in Figure 8b. Since the structure of Network 1 was relatively simple, the network required several iterations to obtain the desired accuracy situation, so setting the number of network iterations to 800 was superior to other combinations of the parameter setting.

Network 2 added a feature return module to the backbone network, consisting of a stack of residual modules to store and utilize features. The configuration of Network 2 is shown in Figure 9a, in which the back propagation module was added. Network 2’s test results for each parameter are presented in Figure 9b. From the group of data in Figure 9, we can notice that the training efficiency had been improved more significantly. In total, 500 iterations of the network were at the same level as the 800 iterations, but the network with 800 full iterations could learn more refined features and had higher accuracy.

Network 3 was built based on Network 2 by adding the standard Transformer model to the backhaul module to perform secondary processing of the stored backhaul features. Figure 10a shows the configuration of Network 3, and Figure 10b illustrates the test results for each parameter of Network 3. In Network 3, the Transformer model was added to process the stored features secondarily, so that better accuracy values were available when 500 iterations were completed. As the iterations proceeded, the model showed some oscillation, so the effect of 800 iterations was not as good as 500 iterations.

Network 4 was built on Network 3 by changing the Transformer model to the lightweight version. Figure 11a represents the configuration of Network 4, and the experimental results of each parameter of Network 4 are shown in Figure 11b. Network 4 had the same structural settings as Network 3, so the two models were similar in terms of the settings of the hyperparameters and had better accuracy values when 500 iterations were completed. In the later stages, as the iterations proceeded, the model showed some oscillations; therefore, the effect of 800 iterations was inferior to that of 500 iterations.

Network 5 added the Kalman filter to the location information storage module on top of Network 3 to predict the location information for the next moment. The configuration of Network 5 is shown in Figure 12a. For each parameter in Network 5, the test results are shown in Figure 12b. In the comparison of the hyperparameter settings of Network 5, the settings of the fourth group were more suitable for this network. Combining the structural design and test comparison of Network 3 and Network 4, we could conclude that Network 5 was similar to them, and the optimal results could be obtained by 500 times of the overall iteration training.

Network 6 added the Kalman filter to the location information storage module on the basis of Network 4 to predict the location information for the next moment. The configuration of Network 6 is presented in Figure 13a, while Figure 13b showed the experimental results for each parameter of Network 6. The data shown in Figure 13 indicated that Network 6 could be iterated to gain better parameters with the configuration of parameter 4. A large amount of iterations caused the network oscillations at the subsequent time, making all aspects of the performance metrics worse than the fourth set of the hyperparameter settings.

According to the training data depicted in Figure 8b, Figure 9b, Figure 10b, Figure 11b, Figure 12b, and Figure 13b, we could conclude that adding the back propagation module into the network could significantly improve the training efficiency of the network. The number of iterations was very high, which led to oscillations in the network during subsequent training, resulting in a certain decrease in the accuracy. For the backbone network composed of the residual modules alone, the simple structure of the network was less capable of learning the features, and more iterations were needed to ensure the model’s convergence. In summary, the results of the optimal evaluation parameters for each network model were extracted and tested for comparison.

**Figure 14:**
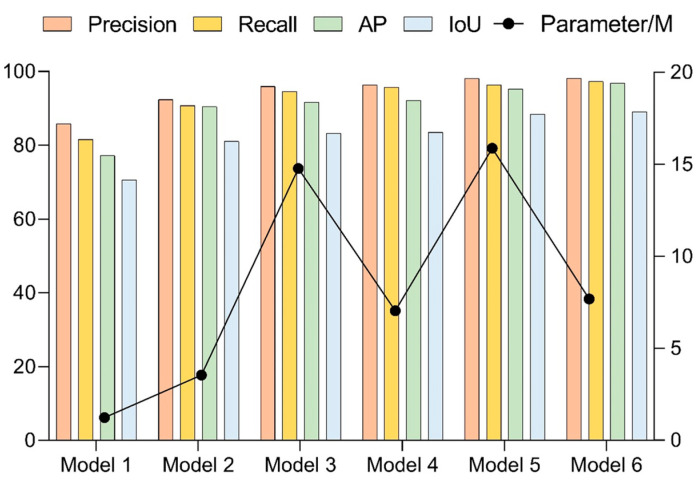
Comparative results of ablation experiments.

The structural configuration of the six networks and the results of the comparative tests are shown in Figure 14 and Table 3.

The data comparison between Network 1 and Network 2 was evaluated by assessing whether the addition of feature storage units could improve the network’s performance. Only the backbone network was included in Network 1, and just six convolutional layers were used for feature extraction. Since the feature information could not be fully extracted, the model performed poorly in both recognition accuracy and localization accuracy and had a low recall index. This indicated that the network had missing detection and could not obtain the coordinates of the target motion trajectory completely. At the same time, due to the poor positioning accuracy, the coordinate information obtained by the network was not referential. Network 2 has more depth in the time dimension because of the storage module that was included, which allows for the reuse of the feature data that was retrieved from the previous frame. With the increase in the detection frames, the feature information extracted by the network was richer, and there were significant improvements in the recognition accuracy and localization accuracy, with only small increases in the network complexity. Although Network 1 and Network 2 have obvious advantages in frame rate, they still cannot meet the requirements for accuracy in practical scenarios. Both Network 3 and Network 4 incorporated the Transformer model in the backhaul module, and the difference between them was that Network 3 used the standard Transformer module, while Network 4 used the lightweight version. By comparing Network 3 and Network 4 with Network 2, the Transformer module performed the secondary processing of the stored features to extract global long-range dependent features, which could complement the local features in the backbone network and could effectively improve the network performance. However, the network complexity of both Network 3 and Network 4 had increased somewhat, compared with Network 2. At the same time, Network 3 and 4 had decreased the frame rate compared with Network 2 to a certain degree. In addition, due to the lightweight Transformer module design of Network 4, its complexity was only half of that in Network 3, and it is also slightly higher than Network 3 in terms of the detection accuracy. Network 5 and Network 6 incorporated the Kalman filter in the return transmission of the position information, which could predict the position information of the sphere in the current frame based on the motion trajectory of the sphere in the preceding frame. The results of comparing Network 5 and Network 6 with Network 3 and Network 4, respectively, showed that the appended Kalman filter could effectively improve the determination accuracy of the position information. In addition, the detection speed of the model can be satisfied with the capturing speed of the high-speed camera.

**Figure 15:**
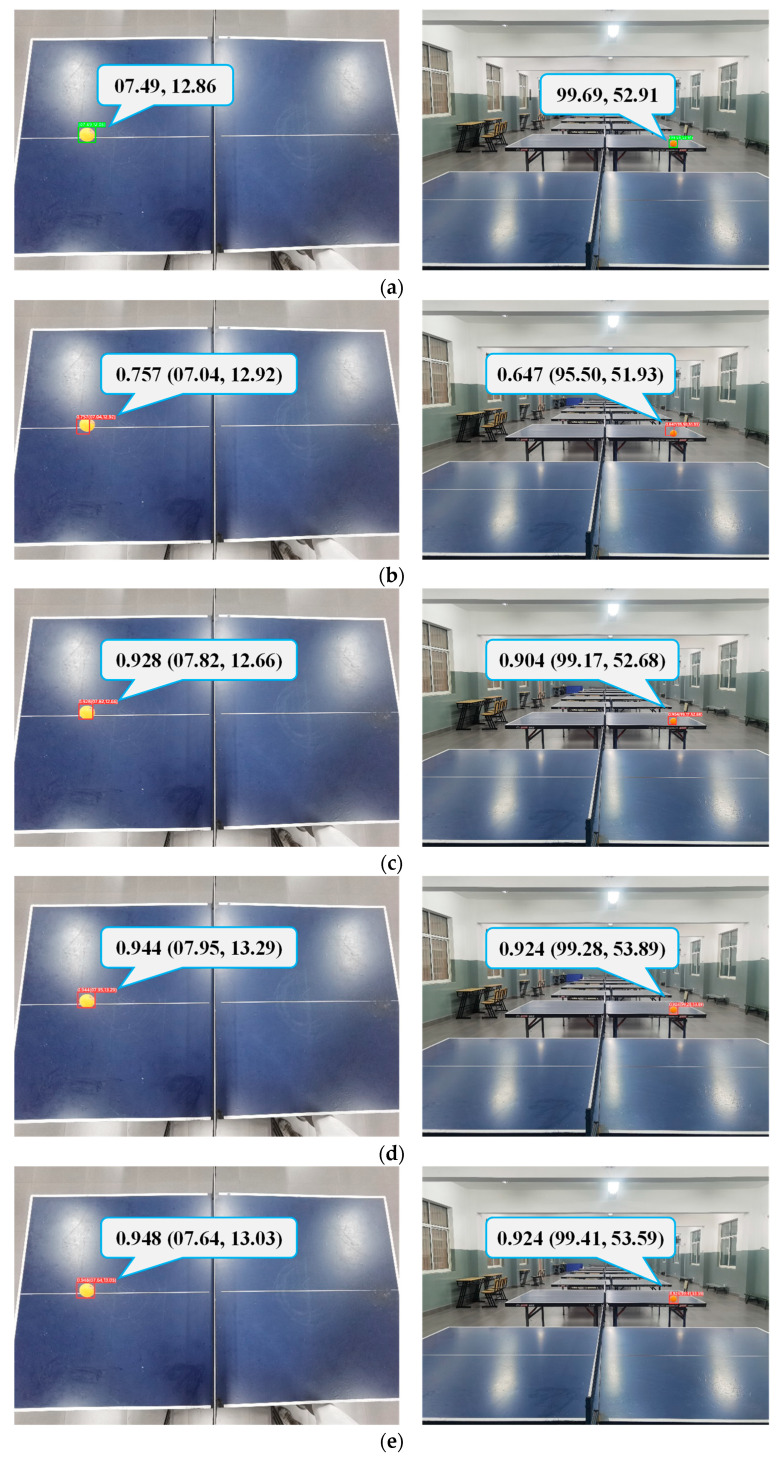

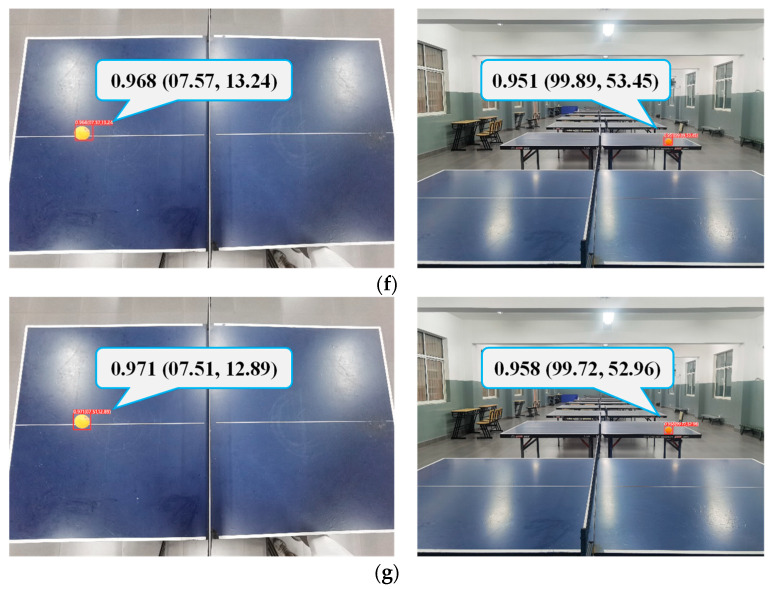
Comparison of target detection results using consecutive frames: (**a**) GroundTruth, (**b**) Network 1, (**c**) Network 2, (**d**) Network 3, (**e**) Network 4, (**f**) Network 5, (**g**) Network 6.

Figure 15 showed that the six network models had large differences in the determination of the target locations for the same frame. The predicted position region of Network 1 was substantially shifted from the actual position region of the target. With adding the back propagation module, Network 2 could roughly frame the target. Network 3 and Network 4 had improved detection accuracy compared with Network 2 after the addition of secondary processing in the transformer model, while the predicted position information had higher conformity with GroundTruth when the Kalman filter was added to Network 5 and Network 6 to determine the position. Meanwhile, for the detection speed of a single frame, the elapsed time of Network 6 was 8.26 ms, which was converted into a frame rate of 121 FPS. This indicated that, even with the high network complexity, the real-time detection using video frames could be achieved in this study.

**Figure 16:**
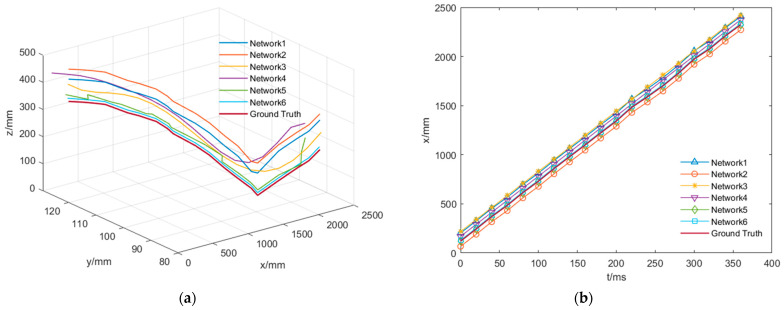

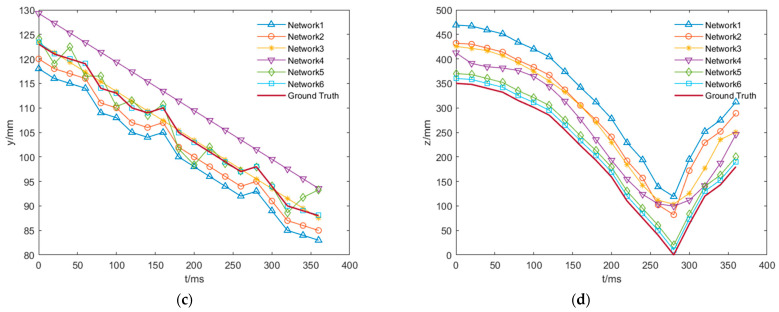
Comparison results of different networks: (**a**) 3D trajectory diagram of the sphere movement, (**b**) trajectory of the sphere’s motion in x-coordinates, (**c**) trajectory of the sphere in y-coordinate motion, (**d**) trajectory diagram of the z-coordinates of the sphere’s motion.

Next, spatial modeling of the position information obtained after the horizontal shot over the vertical shot was performed to determine the sphere motion trajectory. In this control experiment, GroundTruth samples were acquired by depth camera shots, and the rest of the motion trajectories were gained by the method described in this article. In Figure 16, the comparisons are displayed, respectively.

Figure 16 visualizes the accuracy of each model in the ablation experiment for detecting the motion trajectory of the sphere. From the depiction of the trajectories in the overall dimension and in each coordinate dimension, the same conclusion as the results of the ablation experiment could be drawn. The optimized Network 6 could detect the sphere position more accurately, and the obtained trajectory map was also much closer to the real trajectory of the sphere.

### 4.3. Cross-Sectional Comparison Experiments

**Figure 17:**
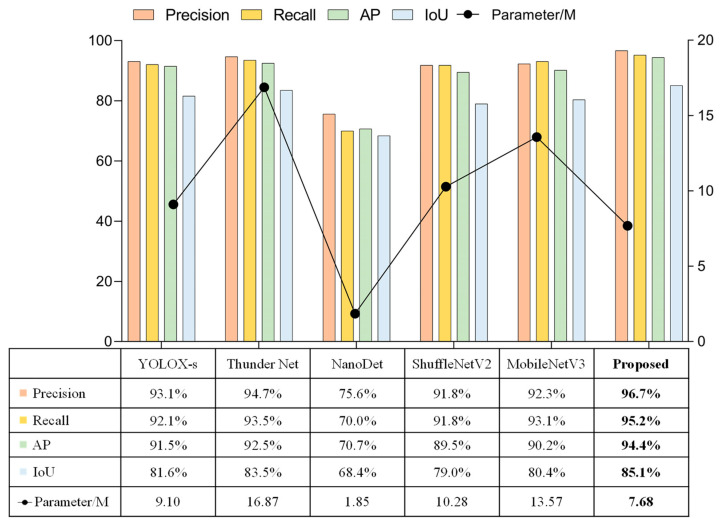
Experimental results of horizontal table capturing.

**Figure 18:**
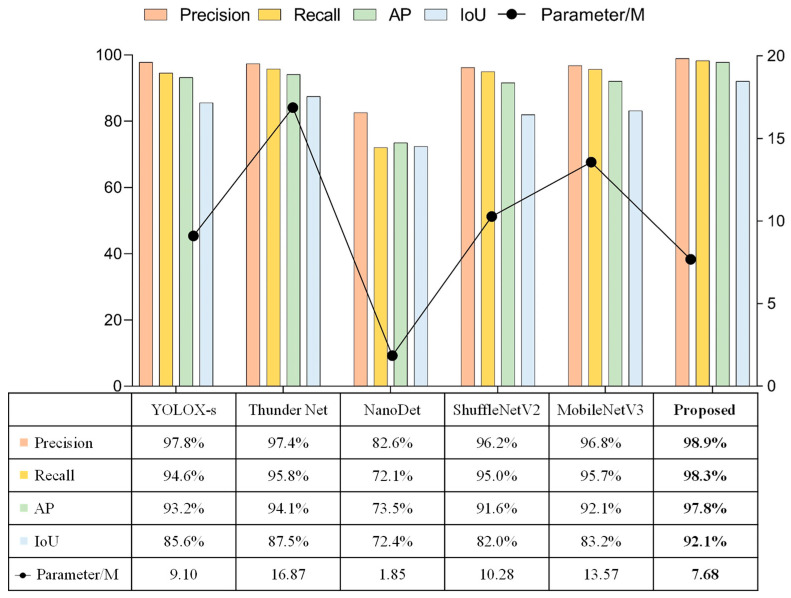
Experimental results of vertical table capturing.

**Figure 19:**
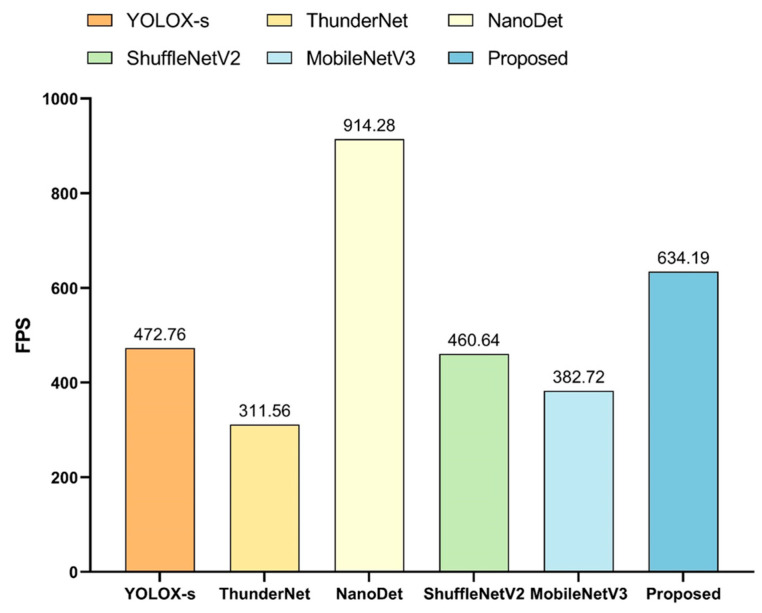
Comparison of detection speed of different networks.

The model proposed in this paper performed cross-sectional performance comparison experiments with lightweight detection networks that were currently widely used in multiple fields. The selected comparison networks included the lightweight one-stage detection model YOLOX [91], the two-stage lightweight detection network ThunderNet [92], the ultra-lightweight Anchor Free detection network NanoDet [93], and detection networks using the lightweight backbone networks, such as ShuffleNetV2 [94] and MobileNetV3 [95], combined with the detectors proposed in this paper. The comparison experiments were subdivided into the detection of targets when shooting horizontally versus vertically, and the results of the comparison tests were shown in Figure 17, Figure 18, and Figure 19, respectively.

All these experiments showed that NanoDet was the best in terms of lightness and frame rate, followed by the network proposed in this paper. However, NanoDet performed the worst in terms of the detection accuracy. Compared with YOLOX-s and ThunderNet, the proposed network had advantages in both the detection accuracy and lightweight. The main difference between YOLOX-s and ThunderNet was the disparity in the localization accuracy. Comparing the proposed network with the detection networks using ShuffleNetV2 and MobileNetV3 as the main networks, the main differences were in the localization accuracy and model complexity, and the proposed model had higher localization accuracy and fewer network parameters. ShuffleNetV2 and MobileNetV3 networks were mainly used to reduce the number of network parameters by deep separable convolution, but the number of network layers could not be reduced significantly. Therefore, this drawback makes their detection frame rates unable to meet the camera’s capturing frame rate, and the practicality was poor.

In summary, considering the three aspects of recognition accuracy, localization accuracy, and network complexity, the network proposed in this paper had great advantages and was well-suited to the scenario described in this work.

### 4.4. Discussion

According to the aforementioned experimental findings, the network developed in this research was more practical in terms of both the detection speed and accuracy, when compared with previous lightweight networks of the same kind.

The proposed network utilized a feature reuse module to achieve the high-accuracy and fast detection of spheres. The feature reuse module was responsible for storing and reusing features, and by embedding it in the network layers, it allowed the network to extend in time dimension, meaning that even a shallow network can achieve the same detection performance as a deep network after sufficient iterations. Additionally, this module also allowed for the storage and prediction of positional coordinate information, providing more accurate target region pointing, compared to the anchor-based methods.

In the above experiments, the improvement scheme and the multi-type lightweight network were evaluated in terms of the detection accuracy and speed (model complexity). According to the ablation experimental results, the rationality of each module designed and its adaptability to the proposed overall network can be demonstrated. The novelty and practicality of the network structure designed in this paper could be derived from the experimental results of the cross-sectional comparison. Compared with the lightweight design of the convolution kernel, we used the lightweight design of the whole network to complete the lightweight design without losing the performance of the convolution kernel.

In summary, in order to realize the lightweight of the network, this article relied on the high similarity of images captured by high-speed cameras to store and reuse features. Moreover, the network also had a high accuracy level of prediction, which was practical and forward-looking in this task.

## 5. Conclusions and Future Work

In order to obtain the motion coordinates of table tennis ball relative to the table during the match, a detection network was designed for real-time detection using videos of a high frame rate. The network relied on the feature storage and return module designed in this paper, which combined the detection accuracy and lightweight features. The storage and return module in each network layer could retain the feature information extracted from the previous frame and pass it to the input of the current network layer through the lightweight Transformer module for the secondary processing of the features. Next, the position information output by the regressor in the detection module was stored. Then, the Kalman filter was used to predict the position information of the previous frame at the next moment, and the predicted position coordinates were transmitted back to the backbone network and combined with the feature map, so that the target position predicted by the current detector was more accurate. Overall, through efficient feature reuse, the network still had high accuracy after reducing the number of network layers, combining detection speed and detection accuracy. In addition, it was experimentally verified that the average detection accuracy of table tennis in horizontal and vertical views was as high as 96.8% and the average localization accuracy is 89.1%, while the number of parameters of the model was only 7.68 MB, which indicated the high practicality of the network.

However, this method relied on the feature extraction results of the previous frame and was based on the detection results of the previous frame to fine-tune and predict the position of the table tennis ball in the current frame. Therefore, the detection accuracy at the beginning of the frame was not ideal. In the next step, a two-stage network would be designed, in which the high-level network would initially adopt frame-skipping detection to abandon a certain detection speed for obtaining high-quality feature maps and transferring them to the low-level network. Thus, the low-level lightweight network could obtain high-quality feature maps for the efficient reuse within a few frames. In the meantime, in later research, an analysis system will be added to the network to decompose and obtain the opponent’s common attacking style, defensive style, and weak points based on the trajectory data of table tennis. The network will be dedicated to providing more effective and direct information for the players to analyze the opponent’s game video before the match, and to distinguish the opponent’s hitting style and trajectory in real time for coaches during the match.

## Figures and Tables

**Table 1:** Analysis of the previous table tennis trajectory tracking method.

	Advantages	Disadvantages	Results
Literature [7]	The ball flight model was established, and the BP pattern recognition classifier was employed, to identify patterns based on the predicted flight path.	The prediction ability of sphere trajectory was weak and the deviation was serious.	The accuracy of the trajectory prediction algorithm of the ball was above 92%.
Literature [8]	A DCNN-LSTM (Deep Convolutional Neural Network Long Short-Term Memory) model was mainly used to improve the real-time performance of motion characteristics extraction through deep enhancement. In the network structure, DCNN was responsible for tracking and identifying objects, and the LSTM algorithm was responsible for predicting the trajectory of the ball.	The model did not have enough robustness and anti-interference ability and it was easy to lose the tracking target in the scene with complex background.	The mean and standard deviation of the error were 36.6 mm and 18.8 mm, respectively.
Literature [9]	This article learned the parabolic trajectory of table tennis on the pivot of table tennis by building a neural network and realized the consequence of table tennis trajectory.	The model learned and predicted the overall trajectory, and the fit was good on the whole, but there were some deviations in the details.	The average error and standard deviation were 36.6 mm and 18.8 mm.
Literature [10]	The automatic detection model was constructed by integrating a compensation fuzzy algorithm with a recursive neural network, resulting in a compensation fuzzy neural network algorithm.	The model was complex and needed to rely on high performance equipment for reasoning.	The precision of motion trajectory prediction improved, as the quantity of input data increased. A prediction error of less than 40 mm was achieved when utilizing 30 pieces of input data.
Literature [11]	A table tennis target trajectory tracking algorithm combining machine vision and scale conjugate gradient was proposed to judge the rotating state of table tennis.	For the ball with fast hitting speed, it could not track the target effectively, and the target loss rate was higher.	The mean square error of the three-axis error of the neural network in this paper was 4.66.

**Table 2:** Hyperparameter settings.

	Epoch	Learning Rate (LR)	Momentum	Weight Decay
1	300	0.0001	0.90	0.0005
2	300	0.0001	0.95	0.0005
3	500	0.0010	0.90	0.0005
4	500	0.0001	0.95	0.0005
5	700	0.0010	0.90	0.0005
6	700	0.0001	0.95	0.0005

**Table 3:** Comparative results of ablation experiments.

	Backbone Network	Return Module	Transformer Module	Lightweight Transformer Module	Kalman Filtering	Precision	Recall	AP	IoU	Parameter/M	FPS
1	√					85.8%	81.6%	77.2%	70.6%	1.23	935.67
2	√	√				92.4%	90.8%	90.5%	81.1%	3.54	906.13
3	√	√	√			96.0%	94.6%	91.7%	83.3%	14.77	348.73
4	√	√		√		96.4%	95.8%	92.2%	83.5%	7.04	677.45
5	√	√	√		√	98.1%	96.4%	95.3%	88.5%	15.87	343.91
6	√	√		√	√	98.2%	97.3%	96.8%	89.1%	7.68	634.19

## Data Availability

Not applicable.
